# End-hole Versus Microvalve Infusion Catheters in Patients Undergoing Drug-Eluting Microspheres-TACE for Solitary Hepatocellular Carcinoma Tumors: A Retrospective Analysis

**DOI:** 10.1007/s00270-018-2150-6

**Published:** 2019-01-11

**Authors:** Joseph J. Titano, Aaron M. Fischman, Arnav Cherian, Madeline Tully, Lance L. Stein, Louis Jacobs, Raymond A. Rubin, Michael Bosley, Steve Citron, Dean W. Joelson, Roshan Shrestha, Aravind Arepally

**Affiliations:** 1 Department of Interventional Radiology, Mount Sinai, New York, NY USA; 2 Transplant Institute, Piedmont Healthcare, Atlanta, GA USA; 3 Division of Interventional Radiology, Piedmont Healthcare, 1984 Peachtree Road, Suite 505, Atlanta, GA 30309 USA; 4 Division of Pathology, Piedmont Healthcare, Atlanta, GA USA

**Keywords:** Locoregional therapy, Liver transplantation, Liver explant, Hepatocellular tumor pathology, Anti-reflux catheter

## Abstract

**Introduction:**

Pre-transplant locoregional therapy for hepatocellular carcinoma (HCC) during bridge-to-transplant impacts recurrence and survival rates following liver transplantation. Optimizing the effectiveness of transarterial chemoembolization (TACE) in this population is imperative, and microvalve infusion catheters offer a means of such improvement.

**Methods:**

All treatment-naive patients with solitary HCC tumors < 6.5 cm who underwent drug-eluting microspheres (DEM) TACE between 04/2015 and 08/2017 were retrospectively reviewed. Eighty-eight included patients underwent DEM-TACE with either standard end-hole catheters (EH) or microvalve infusion catheters (MVI). The EH (*n* = 70) and MVI (*n *= 18) cohorts had similar baseline tumor size, laboratory values, and tumor etiologies.

**Results:**

Initial objective response rates were significantly higher in MVI vs. EH (100% vs. 76.5%, *p *= 0.019). There was no difference in adverse events between groups (*p* = 0.265). MVI patients exhibited lower AST (*p *= 0.003) and ALT (*p *= 0.044) at 6 months. Blinded pathological analysis of explanted livers showed greater concentrations of microspheres within the tumor relative to the surrounding tissue in MVI explants (88.7 ± 10.6%) versus the EH explants (55.3 ± 32.7%) (*p *= 0.002). There was significantly higher percentage tumor necrosis in the MVI group (89.0 ± 2.2%) compared with the EH group (56.1 ± 44.5%) (*p *= 0.006).

**Conclusion:**

In this retrospective study of a single-center cohort, DEM-TACE procedures with MVI were associated with improved tumor response, increased deposition of microspheres within tumor tissue, and higher percentage tumor necrosis at explant relative to those performed using EH catheters.

**Electronic supplementary material:**

The online version of this article (10.1007/s00270-018-2150-6) contains supplementary material, which is available to authorized users.

## Introduction

Transarterial chemoembolization (TACE) is effective in bridging patients to liver transplantation for patients with hepatocellular carcinoma (HCC) [1–4]. As typical patients with the diagnosis of HCC are undergoing evaluation for orthotopic liver transplantation [5], maximizing the effectiveness of bridging therapies is an essential component of a liver transplantation program.

Microvalve infusion catheters (MVI) allow operators to temporarily modulate pressure within a vessel during infusions to promote distal penetration in animal studies while preventing retrograde flow to uninvolved regions of liver parenchyma [6]. MVI catheters have also shown the ability to increase uptake of MAA particles delivered in tumors and to improve homogeneity of particle distribution in a swine model, and finally, improved tumor response rates in HCC tumors beyond UNOS transplantation criteria have been described [7–10]. The aim of the current study is to compare imaging response rates, local tumor recurrence rates, hepatotoxicity, explant pathology, and microsphere distribution in and around tumors after drug-eluting microsphere (DEM) TACE performed utilizing standard end-hole (EH) catheters versus MVI catheters in solitary tumors. Thus, the underlying hypothesis was that the use of MVI results in a higher rate of response (as demonstrated by mRECIST) than conventional EH catheters in patients with solitary tumors.

## Materials and Methods

### Patient Selection and Clinical Course

**Fig. 1:**
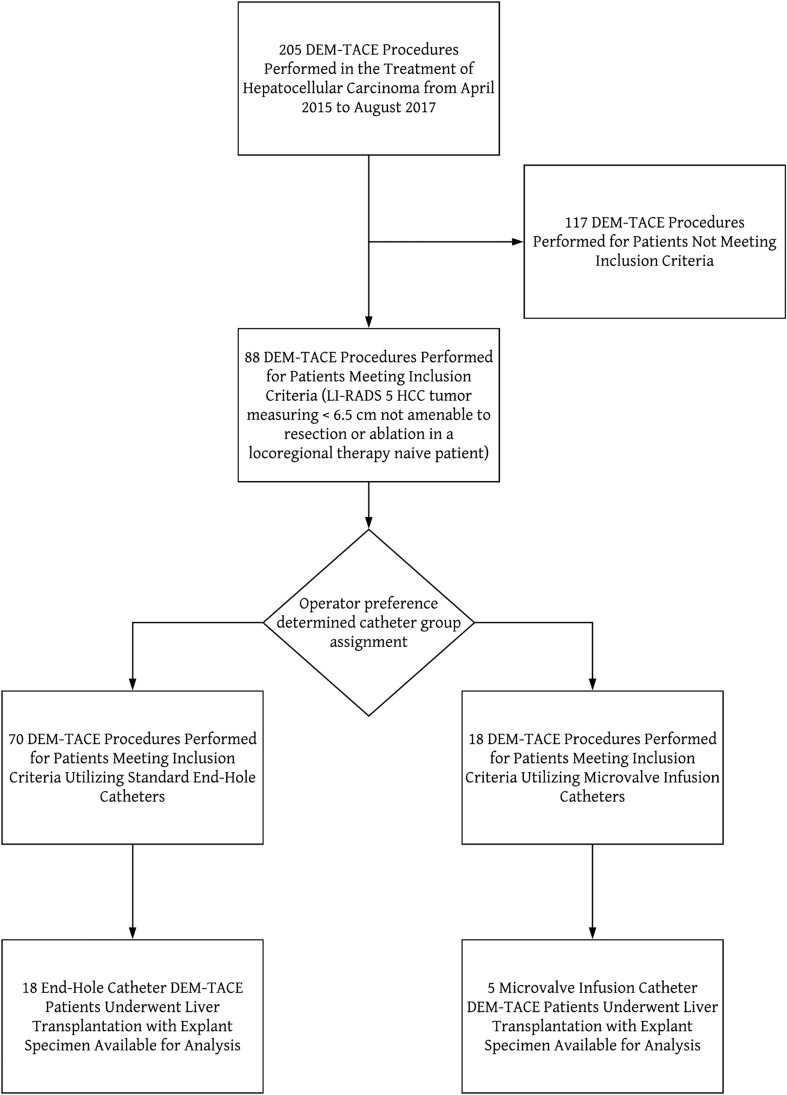
Flowchart demonstrating the derivation of the end-hole and microvalve infusion cohorts

The local institutional review board approved this retrospective, single-center study (Piedmont Healthcare IRB 220165). The study period extended from April 2014 to August 2017. Data were obtained through searching the Epic electronic medical record system (EPIC Inc, Verona, WI). Inclusion criteria for this retrospective analysis were included as follows: treatment naïve, solitary, Liver Imaging Reporting and Data System (LI-RADS) 5 HCC tumor, measuring less than 6.5 cm, tumor not amenable to surgical resection or ablation, treated with DEM-TACE [11]. Patients were excluded from the study if their tumors were previously treated with any other locoregional therapies such as yttrium-90 transarterial radioembolization (Y90) or radiofrequency ablation (RFA) (Fig. 1).

A retrospective review of the medical records was conducted to identify the etiology of liver disease, Child–Pugh score, tumor size, total number of TACE procedures, including repeat TACE interventions and other subsequent locoregional therapies.

All patients underwent full history and physical examination prior to therapy. Operators reviewed each patient’s functional status, laboratory values, and cross-sectional imaging prior to DEM-TACE. Patients considered for treatment with DEM-TACE were all Barcelona Clinic Liver Cancer (BCLC) stage 0 or A, had no imaging evidence of macrovascular invasion (based on contrast-enhanced CT or MRI), and had a total bilirubin level less than 3.0 mg/dL. Patients underwent laboratory assessment 1 month prior to initial DEM-TACE and again within 1 week of follow-up imaging. Adverse events were categorized according to the Common Terminology Criteria for Adverse Events (CTCAE) version 4.03.

Between 4 and 8 weeks post-treatment and then at 3-month intervals thereafter, patients underwent follow-up imaging with either contrast-enhanced, multiphase CT or MRI. Board-certified radiologists (average experience 20 years, range 8–25 years) conducted blinded review of follow-up imaging by applying Modified Response Evaluation Criteria in Solid Tumors (mRECIST) [12].

### TACE Technique

Patients underwent TACE treatments after providing written informed consent. Operators inserted a 5-Fr guide catheter into the common femoral artery and subsequently performed angiographic surveys of the celiac axis and superior mesenteric artery. Digital subtraction angiography was also performed after selective catheterization of the proper, right, and/or left hepatic arteries. Operators then identified tumor-feeding vessels and selected them with a coaxially placed standard EH microcatheter (Renegade HI-FLO, Boston Scientific, Natick, MA) or MVI catheter (Surefire Infusion System, Surefire Medical, Westminster CO). The operators were familiar with both systems and made the choice of catheter selection during each procedure.

DEM-TACE was performed with 100–300 µm microspheres (LC Beads; BTG International Ltd, UK) loaded with 75–150 mg of doxorubicin. The administered doses of the chemotherapy agent were adjusted in patients with liver or renal dysfunction, leukopenia, and thrombocytopenia.

In procedures performed with EH, operators performed administration of the microspheres until the maximum 150 mg doxorubicin dose was delivered or stasis was achieved. Stasis was defined as the absence of flow into the target vessel with the development of reflux visible on angiography.

When utilizing MVI, operators delivered drug-eluting microspheres until one of the following endpoints was achieved: leaching of contrast medium retrograde through the expandable tip, development of an intrahepatic collateral vessel leading away from the target tumor, or visualization of the portal vein.

### Histopathologic Evaluation

A board-certified pathologist (10 years experience) conducted blinded review of the liver explant specimens to assess the extent of tumor necrosis and the distribution of microspheres in and around the treated tumor.

All explanted livers were processed per routine clinical protocol at our institution. The freshly explanted livers were sliced serially at 10-mm intervals. Macroscopically visible neoplastic nodules were evaluated with microscopy after hematoxylin and eosin staining. The percentage necrosis was defined as the volume of necrotic areas divided by the total tumor volume. Percentage on-target microsphere distribution in the tumor was defined as the number of microspheres present within the boundaries of the tumor divided by the total number of microspheres in the slides with tumor. Additional explant details were recorded including tumor size, tumor location, and tumor grade.

### Statistical Analysis

Categorical variables are reported as number (percentage). Continuous data are reported as mean (standard deviation) or as median (interquartile range [IQR]). Categorical variables were compared through either the Chi-square test or Fisher’s exact test. Continuous variables were compared through independent samples *t* test or Kruskal–Wallis test where appropriate. Imaging response outcomes between groups were compared using logistic regression. A Cox proportional hazards model was utilized to analyze factors impacting disease progression. Statistical analysis was performed using SPSS statistics package for Windows, version 24.0 (IBM, Armonk, NY). A *p* value < 0.05 was considered significant.

## Results

Over the study period, 88 patients met the inclusion criteria of this retrospective review. Baseline characteristics are presented in Table 1. At baseline, there was no difference between the groups in patient sex (*p* = 0.749), tumor size (*p* = 0.78), incidence of hepatitis C (*p* = 0.089), incidence of hepatitis B (*p* = 0.586), incidence of nonalcoholic steatohepatitis (NASH) (*p* = 0.922), or incidence of alcohol liver disease (*p* = 0.863). There were two instances of crossover in which patients initially treated with EH catheters were subsequently treated with MVI catheters. Therefore, these two patients were removed from the mRECIST review evaluation at 4–8 months and also were removed from the local recurrence rate analysis at 4–8 months. Two patients in each group were later treated with sorafenib therapy following disease progression, which is not significantly different between the groups (*p* = 0.387). Two patients in the EH group did not have follow-up imaging or laboratory data available.

**Table 1:** Baseline patient demographics and procedure characteristics

Characteristics	EH	MVI
Patients	70	18
Sex (M/F)	56/14	15/3
Mean age	62 (41–81)	61 (55–74)
BCLC stage 0/A	70	18
Etiology of liver disease		
Hepatitis C	46 (65.7%)	8(44.4%)
Hepatitis B	3 (4.3%)	3 (16.7%)
Alcohol abuse	29 (41.4%)	8 (44.4%)
Hepatitis C + alcohol	21 (30%)	5 (27.8%)
ECOG 0	70	18
Treatment naïve	70	18
Mean tumor size (cm)	3.1 (± 1.2)	3.1 (± 1.1)
Watershed tumors	10 (14.3%)	2 (11.1%)
AFP > 400	5 (7.3%)	1 (6.6%)
Total bilirubin	1.4	1.5
Mean doxorubicin dose (mg)	81.5 (10–225)	64 (25–125)

Data are presented as number (range), number (percentage), or mean (± standard deviation) where appropriate. *EH* end-hole catheter, *MVI* microvalve infusion catheter, *M* male, *F* female, *BCLC* Barcelona Clinic Liver Cancer, *ECOG* Eastern Cooperative Oncology Group, *AFP* alpha-fetoprotein. Watershed tumor was defined as a tumor located in segment 4a or 4b

### Clinical, Laboratory, and Imaging Outcomes

There was no significant difference between chemotherapy dose delivered to the tumor between the EH group (81.4 ± 41.8 mg) and the MVI group (62.2 ± 30.6 mg) (*p* = 0.072). The numbers of follow-up treatments required within the first 6 months after initial DEM-TACE were not significantly different between the two groups (*p* = 0.427). In the EH group, 13 (18.6%) patients received subsequent TACE, four (5.7%) patients received Y90, and three (4.3%) patients received RFA after the initial DEM-TACE procedure. In the MVI group, no patients received subsequent TACE or Y90, and only one (5.6%) patient received RFA after the initial DEM-TACE procedure.

Comparison of imaging response at initial follow-up revealed that the incidence of mRECIST objective response (OR) was significantly greater in the MVI group (100%) relative to the EH group (76.5%) (*p* = 0.019). At 1 month, the CR in the MVI group was at 66.6% and in the EH group was 50.0% (*p* = 0.200). At 6 months post-treatment, 35.4% of patients in the EH group demonstrated local progression of disease on follow-up imaging vs. 14.3% in the MVI with no statistically significant difference between the groups (*p* = 0.413).

At baseline, alpha-fetoprotein (AFP) tumor marker values showed no significant difference between the two groups (*p* = 0.131). At 1 month post-treatment, the percentage change in AFP from baseline (in patients with AFP ≥ 400 ng/ml) was not significantly different between the two groups (EH: − 16% ± 39%, MVI: − 40% ± 33%, *p* = 0.090). However, at 3 months post-treatment, there was a significant difference in percentage AFP change from baseline between the two groups (EH: 22% ± 170%, MVI: − 36% ± 35%, *p* = 0.040).

There was no difference in serum total bilirubin levels between the two groups at baseline (*p* = 0.380), at 1-month follow-up (*p* = 0.758), at 3-month follow-up (*p* = 0.135), or at 6-month follow-up (*p* = 0.614).

There was no difference in aspartate aminotransferase (AST) levels between the two groups at baseline (*p* = 0.298), at 1-month follow-up (*p* = 0.596), or at 3-month follow-up (*p* = 0.222). At 6-month follow-up, lower AST levels were observed in the MVI group (29.6 ± 18.7 U/L) compared to the EH group (84.5 ± 149.5 U/L) (*p* = 0.017).

The alanine aminotransferase (ALT) levels at baseline were more favorable in the MVI group (30.5 ± 15.6 U/L) compared to the EH group (53.1 ± 50.1 U/L) (*p* = 0.003). But there was no difference in ALT levels between the two groups at 1-month follow-up (*p* = 0.201) or at 3-month follow-up (*p* = 0.336). At 6-month follow-up, however, the ALT levels were again more favorable in the MVI group (ALT = 26.9 ± 22.0 U/L) compared to the EH group (ALT = 81.9 ± 234.1 U/L) (*p* = 0.044). A complete set of laboratory values for the cohorts are summarized in Supplementary Table 1.

There was no statistically significant difference in major complication rates between the treatment cohorts (*p* = 0.265). One (1.4%) patient in the EH group developed a biloma. Three patients in the EH group (4.3%) compared with two patients (11.1%) in the MVI group developed portal vein thrombus following DEM-TACE without significant difference between the cohorts (*p* = 0.265).

### Explant Analysis

**Fig. 2:**
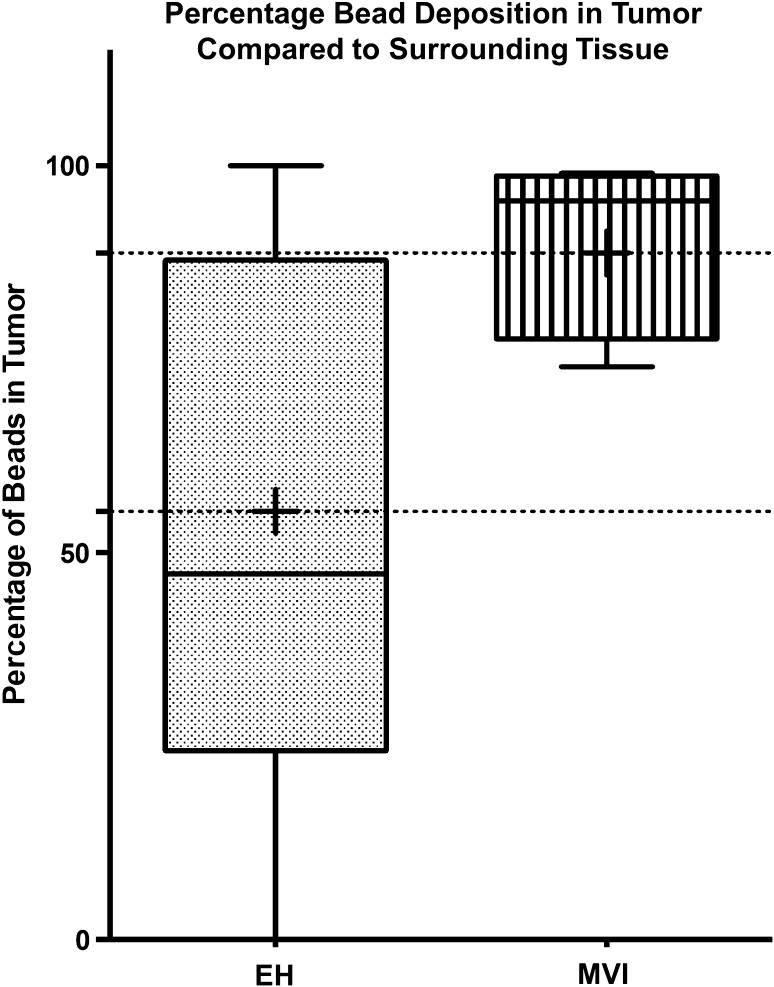
Percentage drug-eluting microsphere deposition within tumor. Comparison of the percentage of drug-eluting microspheres noted within tumor tissue relative to surrounding tissue revealed significantly more microsphere deposition in the MVI explants (*p *= 0.002)

Twenty-three patients (18 EH, 5 MVI) received a liver transplant during the study period with a median waiting time of 275.9 (± 134.2) days in the EH group compared to 173.2 (± 265.7) days in the MVI group (*p* = 0.985). Histopathologic evaluation of the explanted liver specimens revealed that the target tumor sizes were larger in the MVI group (3.2 ± 3.4 cm) compared to the EH group (2.2 ± 2.6 cm) (*p* = 0.0357). Greater on-target distribution of microspheres was found in the MVI explant specimens, with an average of 88.7 ± 10.6% of the microspheres found in the tumor (intratumoral) vs. surrounding tissue, compared to 55.3 ± 32.7% in the EH group (*p* = 0.002). Graphical representation of the microsphere concentrations is presented in Fig. 2.

**Fig. 3:**
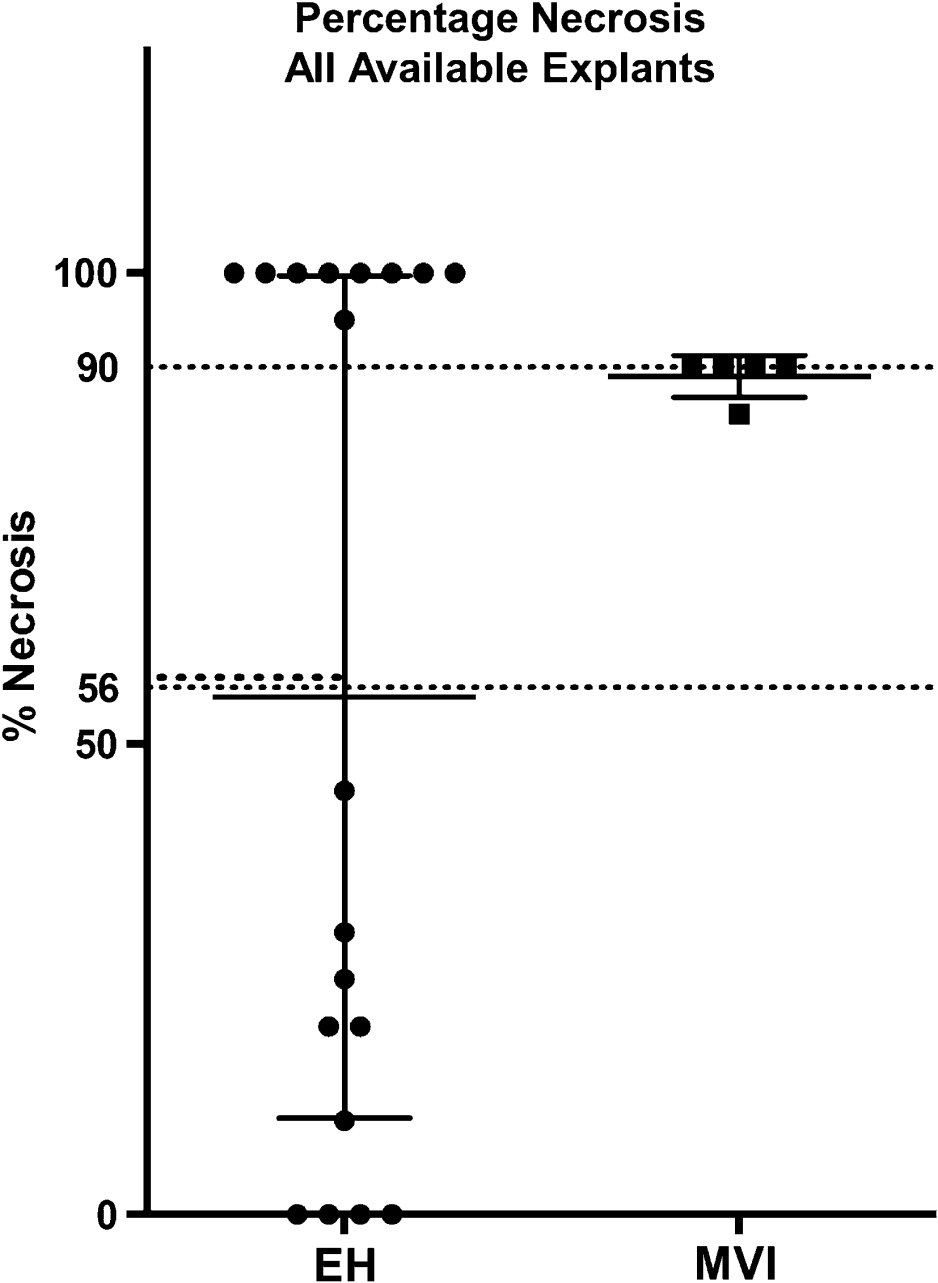
Percentage tumor necrosis: all explants. Including all available explants—even those of patients who underwent subsequent locoregional therapies—in a comparison of explant target tumor necrosis, MVI specimens showed greater percentage necrosis relative to EH specimens (*p *= 0.006)

**Fig. 4:**
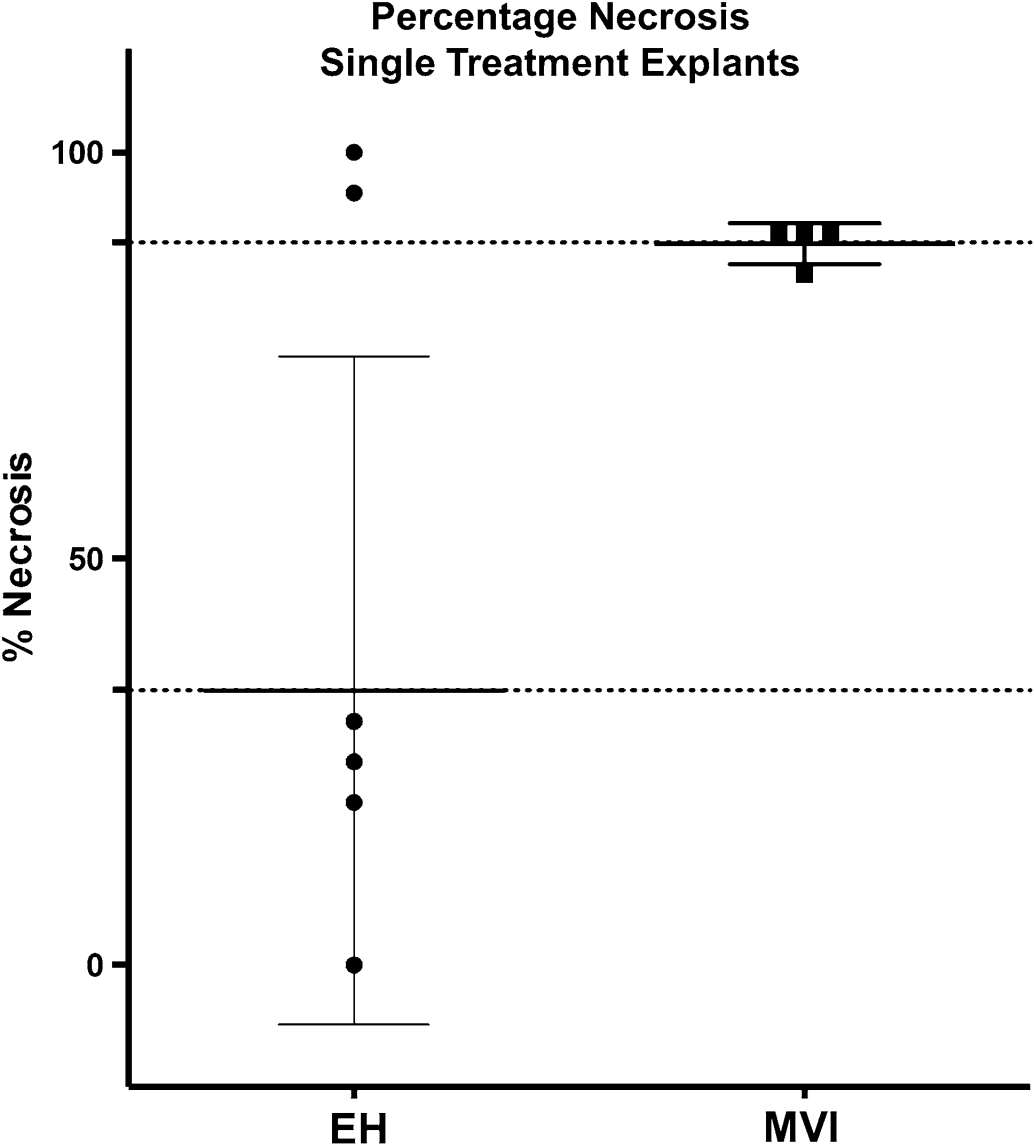
Percentage tumor necrosis: single treatment tumors. Excluding the explants of patients who underwent subsequent locoregional therapies from a comparison of explant target tumor necrosis, MVI specimens showed greater percentage necrosis relative to EH specimens (*p *= 0.026)

Target tumor necrosis percentage was also greater in the MVI liver specimens (89.0 ± 2.2%) relative to the EH liver specimens (56.1 ± 44.5%) (*p* = 0.006). This significant difference in tumor necrosis rate remained after excluding patients who received subsequent therapies prior to transplantation (EH: 3 TACE, 3 Y90, MVI: 1 TACE, 0 Y90) with the average tumor necrosis rate in the MVI explants was 88.8 ± 2.5% vs. 33.8 ± 41.1% in the EH explants (*p* = 0.026). Percentage necrosis data are presented graphically in Figs. 3 and 4.

## Discussion

Under the “HCC Delay” policy instituted by United States OPTN in Oct 2015, candidates with an initial diagnosis of HCC had a mandatory minimum wait time of 6 months for liver transplantation. Thus, with longer wait times for liver transplantation based on this OPTN policy, patients will depend on locoregional therapy in general—and TACE and Y90 TARE—to maintain their disease within transplantation criteria. Recent studies have shown that lack of complete response at initial follow-up after bridge-to-transplant therapy is predictive of drop out [13]. Multiple treatments and lack of complete pathological response after TACE during bridge-to-transplant is also indicative of poor outcomes [14–19]. This growing evidence showing the effect of suboptimal TACE on long-term outcomes post-transplantation elevates the importance of investigating all aspects of the procedure that could impact the effectiveness of TACE in this patient population. Furthermore, the ability to achieve CR in the initial treatment has been correlated with improved survival.

**Fig. 5:**
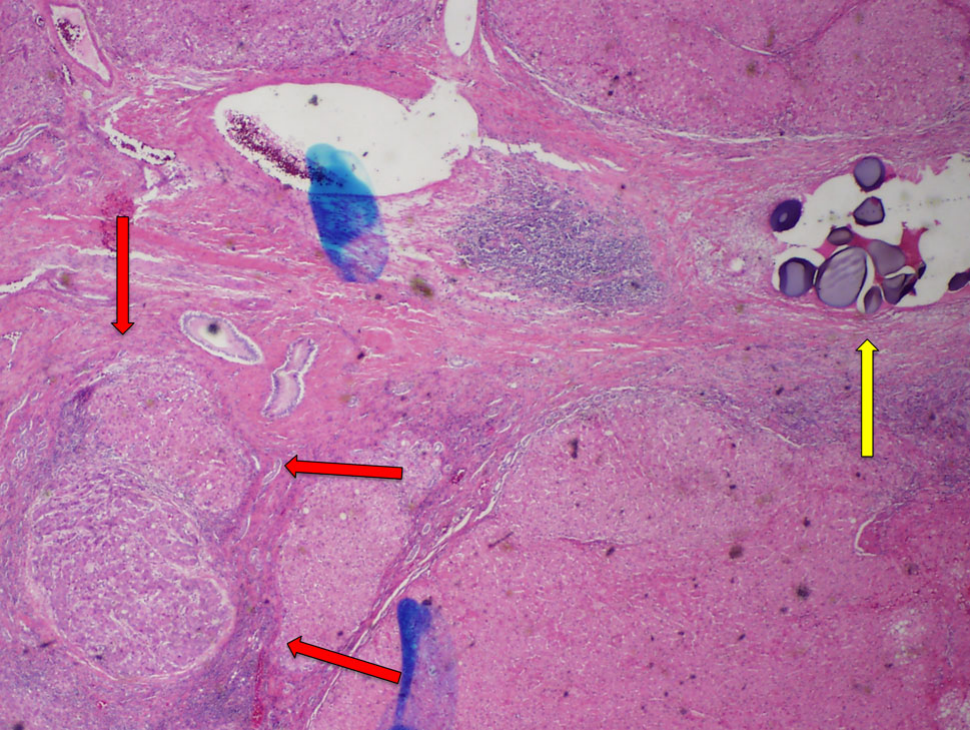
End-hole explant specimen. There is viable tumor tissue (red arrows) identified in this specimen that demonstrated no tumor necrosis. A cluster of drug-eluting microspheres (yellow arrow) is seen distant from the tumor bed

**Fig. 6:**
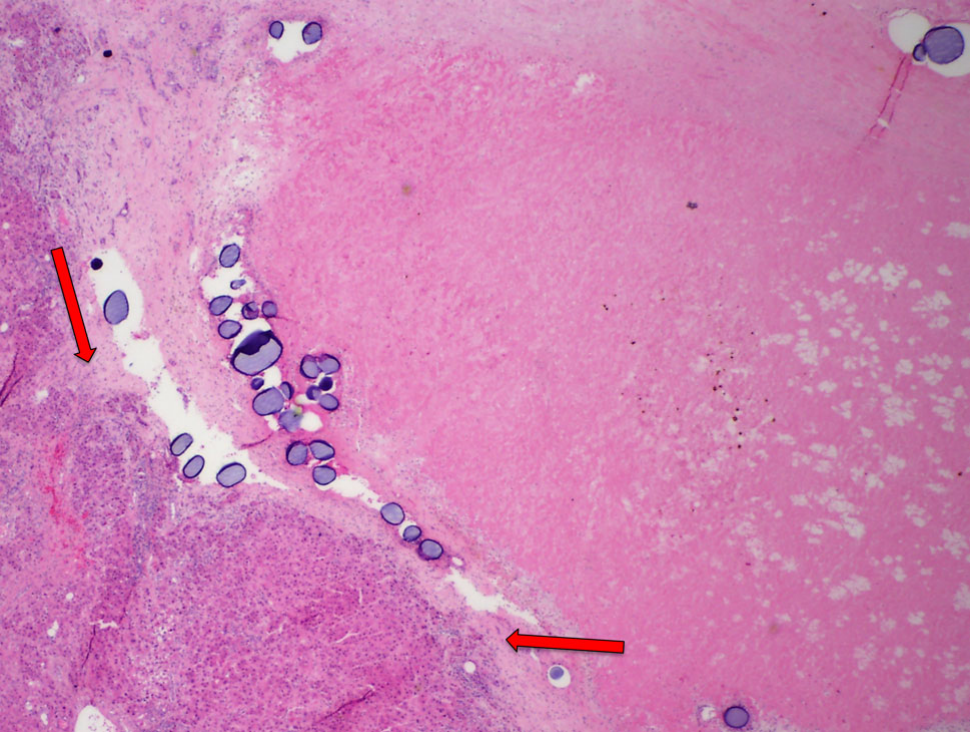
Microvalve infusion explant specimen. There is extensive fibrotic tissue identified compatible with necrotic tumor with a small region of viable tumor tissue (red arrows) still present in this specimen demonstrating 90% tumor necrosis. Several groups of drug-eluting microspheres are also noted within the tumor bed

The current retrospective study shows that the microcatheter used to deliver TACE can have significant effect on tumor response rates and tumor necrosis. There was a statistically significant increase in the ORR in the MVI group; the CR rate at the 1-month follow-up was higher in the MVI group but did not reach statistical significance. Finally, the improved tumor response rates found for MVI correlated with improved histopathologic tumor necrosis of explanted livers. Analysis of explanted liver specimens suggests that the greater tumor necrosis rates observed in the MVI group were a result of improved distal penetration and on-target distribution of microspheres delivered to the tumor (Figs. 5, 6).

In the present study, 100% of the explants in the MVI group exhibited ≥ 50% tumor necrosis compared to 71.4% in the EH group. Three other studies that reported percentage tumor necrosis in liver explants after DEM-TACE were identified [20–22]. In these studies, 66.7% [22], 74.2% [21], and 80.3% [20] of the liver explants exhibited ≥ 50% tumor necrosis. These results are consistent with the tumor necrosis percentage for the control group (DEM-TACE with EH) in this study and slightly inferior to the tumor necrosis percentage for the MVI cohort.

While the tumor necrosis rate found in the MVI explants is encouraging relative to previously published studies, caution is advised when evaluating such comparisons as the sample of MVI explants is limited to five specimens. In addition, direct comparison between previously published studies and the current investigation is limited due to sample heterogeneity in other studies stemming from differences in number of tumors treated in each liver, patients’ BCLC stages (spanning BCLC-0 to BCLC-C), treatment type (conventional vs. DEM-TACE), and number of treatments.

The cohort evaluated by Odisio et al. [22] was the most similar to the explants evaluated in the present study in that all included patients were treated with DEM-TACE and 20 of the 23 included patients had solitary HCC lesion. In the Odisio et al. cohort, 66.7% of the explants demonstrated ≥ 50% necrosis, which is similar to the EH group and is less favorable than the result of MVI patients in our study.

The authors hypothesize that the MVI catheters demonstrated greater on-target microsphere deposition within the tumor and increased tumor percentage necrosis through three mechanisms. First, the expandable tip in the MVI catheter acts as a one-way valve that enables operators to preferentially target tumor tissue by eliminating reflux [23]. During TACE performed with EH microcatheters, the infusion is terminated when stasis is achieved. However, this endpoint may be attained before the tumor bed is completely saturated, resulting in suboptimal response rates [10]. The use of the MVI catheter enables operators to continue administering therapy until complete saturation of the target area is achieved. The revised technical endpoints of TACE treatment undertaken with an MVI catheter, such as the appearance of intrahepatic collateral vessels or visualization of portal vein branches via arterioportal shunting, may provide for a more aggressive treatment of target tumors [24, 25].

Next, the expansion of the microvalve may alter the flow dynamics and preferentially direct blood flow into the abnormally hypervascular tumor beds in comparison with the hepatic parenchyma peripheral to the tumor [24–26]. A similar effect has been observed in TACE performed using a balloon catheter (B-TACE) and has been shown to improve tumor accumulation of chemoembolic emulsion, which is associated with improved tumor response [27–30].

The current study also demonstrates that the improved imaging response rates and higher tumor percentage necrosis in the MVI group can be achieved without increasing rates of liver toxicity or complications. Patients who underwent DEM-TACE with MVI had significantly lower AST and ALT at 6 months post-treatment compared with those in the EH group. The preferential delivery of therapeutic agents into target tumor and minimization of delivery to non-target liver parenchyma through the mechanisms proposed above may account for the reduced incidence of hepatotoxicity seen in the MVI group since the normal liver parenchyma adjacent to the target tumor received a lower exposure to the chemotherapeutic agent.

Interestingly, the delivered doxorubicin dose (81.5 mgs) in the EH cohort was substantially greater than that in the MVI cohort (64 mgs). Prior pre-clinical data demonstrated that the use of MVI catheters resulted in a higher efficiency of delivery (in comparison with EH) into the target vessel due to the elimination of reflux. Thus, the end-hole arm in this swine study had approximately 28% delivery of the agent into non-target adjacent vessels due to reflux [6]. Therefore, the authors hypothesize that although a higher dose was delivered in the EH cohort in this study, a portion of this dose likely refluxed into adjacent vascular beds.

Finally, the use of alternative devices such as balloon occlusion microcatheter for TACE has also described alteration in hepatic blood flow from proximal occlusion. In this technique, the cessation of blood flow with balloon inflation enabled reversal of flow in intrahepatic circulation toward the target vascular bed. However, the ability of balloon occlusion system to enable deeper penetration has not been validated nor studied in an explant analysis [30].

There are several limitations to the current study. The retrospective design of the study introduced the potential for biases including selection bias. While the MVI and EH groups were similar in terms of baseline demographics, disease etiologies, tumor characteristics, and laboratory values, the possibility of selection bias remains since there was no randomization. The group sizes were also relatively small, thereby introducing the possibility of type 2 errors in the statistical analysis. In addition, the single-center nature of the study and the focus of the present study on DEM-TACE both limit generalizability of the results to other locoregional therapy treatment options.

The results of the current clinical inquiry warrant further investigation of MVI catheters and their potential to improve the effectiveness of TACE. At present, there is an ongoing multi-center, randomized controlled trial comparing the response rates of MVI vs. EH catheters in patients with HCC within UCSF criteria (DEB-TACE for Hepatocellular Carcinoma, NCT02748161).

## Conclusion

In summary, DEM-TACE procedures performed in this single-center, retrospective study with MVI were safe and were associated with improved tumor response at initial follow-up, with increased deposition of microspheres, and with higher percentage tumor necrosis at explant relative to those performed using EH catheters. Further investigation is warranted—and is currently underway—to establish the role of MVI in optimizing the effectiveness of TACE for treatment of HCC in transplant population.

## Electronic supplementary material

Below is the link to the electronic supplementary material.

Supplementary material 1 (DOCX 15 kb)

## Abbreviations

ALT: Alanine aminotransferase
AFP: Alpha-fetoprotein
MVI: Microvalve infusion catheter
AST: Aspartate aminotransferase
BCLC: Barcelona Clinic Liver Cancer
CTCAE: Common Terminology for Adverse Events
DEB: Drug-eluting bead
DEM: Drug-eluting microsphere
EH: End-hole catheter
HCC: Hepatocellular carcinoma
IQR: Interquartile range
LI-RADS: Liver Imaging Reporting and Data System
MELD: Model of end-stage liver disease
mRECIST: Modified Response Evaluation Criteria in Solid Tumors
OPTN: Organ procurement and transplantation network
RFA: Radiofrequency ablation
TACE: Transarterial chemoembolization
Y90: Transarterial radioembolization with yttrium-90
