# Supplementary material for: End-hole Versus Microvalve Infusion Catheters in Patients Undergoing Drug-Eluting Microspheres-TACE for Solitary Hepatocellular Carcinoma Tumors: A Retrospective Analysis
Source: Cardiovasc Intervent Radiol. 2019 Jan 11;42(4):560–8. doi: 10.1007/s00270-018-2150-6 (PMC6394778; doi:10.1007/s00270-018-2150-6)
Supplement: Supplementary file 1 — Supplementary material 1 (DOCX 15 kb) [file 270_2018_2150_MOESM1_ESM.docx]

**Supplementary Table 1. Laboratory Values Pre- and Post-Treatment**

| **Laboratory Value** | **One Month Prior** | | **p-value** | **One Month Post** | | **p-value** | **Three Months Post** | | **p-value** | **Six Months Post** | | **p-value** |
| --- | --- | --- | --- | --- | --- | --- | --- | --- | --- | --- | --- | --- |
|  | **EH** | **AR** |  | **EH** | **AR** |  | **EH** | **AR** |  | **EH** | **AR** |  |
| Albumin (g/dl) | 3.36 ± 0.57 | 3.43 ± 0.61 | 0.689 | 3.32 ± 0.61 | 3.06 ± 0.58 | 0.222 | 3.41 ± 0.55 | 3.10 ± 0.63 | 0.137 | 3.43 ± 0.56 | 3.45 ± 0.66 | 0.915 |
| AFP (ng/ml) | 230.5 ± 1034 | 52.47 ± 136.9 | 0.131 | -16% ± 39%* | -40% ± 33%* | 0.090 | 22% ± 170%* | -36% ± 35%* | **0.040** | NA | NA | - |
| ALT (U/L) | 53.10 ± 50.08 | 30.53 ± 15.55 | **0.003** | 39.29 ± 24.32 | 29.18 ± 14.21 | 0.201 | 79.58 ± 267.8 | 42.11 ± 22.56 | 0.336 | 81.92 ± 234.1 | 26.90 ± 21.95 | **0.044** |
| Alkaline Phosphatase (U/L) | 115.2 ± 41.10 | 110.3 ± 58.31 | 0.284 | 131.6 ± 48.92 | 105.2 ± 48.00 | 0.090 | 137.4 ± 57.72 | 109.0 ± 23.16 | 0.198 | 143.9 ± 69.99 | 118.1 ± 57.52 | 0.229 |
| AST (U/L) | 71.46 ± 44.80 | 55.00 ± 27.32 | 0.298 | 58.16 ± 41.56 | 53.45 ± 20.67 | 0.596 | 119.5 ± 406.3 | 47.78 ± 23.38 | 0.222 | 84.47 ± 149.5 | 29.60 ± 18.65 | **0.017** |
| GGT (U/L) | 101.3 ± 71.64 | 113.4 ± 189.5 | 0.150 | 75.14 ± 45.79 | 102.2 ± 113.3 | 0.775 | 174.9 ± 178.2 | 107.7 ± 71.66 | 0.422 | 100.4 ± 85.28 | 78.75 ± 88.54 | 0.253 |
| INR | 1.23 ± 0.21 | 1.25 ± 0.26 | 0.776 | 1.27 ± 0.26 | 1.31 ± 0.27 | 0.779 | 1.29 ± 0.38 | 1.36 ± 0.36 | 0.430 | 1.26 ± 0.26 | 1.20 ± 0.15 | 0.614 |
| Total Bilirubin (mg/dl) | 1.37 ± 0.93 | 1.46 ± 0.88 | 0.380 | 1.60 ± 1.28 | 1.73 ± 0.78 | 0.758 | 1.47 ± 1.17 | 2.93 ± 3.96 | 0.135 | 1.42 ± 1.08 | 1.68 ± 1.36 | 0.614 |

AFP = alpha-fetoprotein, ALT = Alanine Aminotransferase, GGT = gamma glutamyltransferase, AST = Aspartate Aminotransferase, INR = international normalized ratio; *AFP is here reported as percentage change from baseline and the AFP percentage change analysis only includes patients with baseline AFP ≥ 400 ng/ml.
